# Replication of Human Norovirus in Human Intestinal Enteroids Is Affected by Fecal Sample Processing

**DOI:** 10.3390/v16020241

**Published:** 2024-02-02

**Authors:** Revati Narwankar, Malak A. Esseili

**Affiliations:** Center for Food Safety, Department of Food Science and Technology, University of Georgia, Griffin, GA 30223, USA

**Keywords:** human norovirus, human intestinal enteroids, replication

## Abstract

Human intestinal enteroids (HIEs) culture is an emerging model for assessing the infectivity of human noroviruses (HuNoVs). The model is based on detecting an increase in HuNoV RNA post-infection of HIEs. HuNoV fecal samples used for HIE infection are traditionally processed by serial filtration. Recently, processing HuNoV fecal samples by serial centrifugation was shown to retain vesicles containing HuNoV. The objective of this study was to investigate whether serially centrifuged fecal samples, RNA extraction kit (QIAamp versus MagMaX) and HIE age (newer versus older) affect HuNoV RNA fold increase in HIE. HuNoV GII.1, GII.4 and GII.6 fecal samples were prepared by serial centrifugation and filtration and the viral RNA in HIE was quantified at 1 and 72 h post-infection (hpi) following RNA extraction and RT-qPCR. The serially filtered GII.1, GII.4 and GII.6 showed successful replication in HIE, resulting in mean log increases of 2.2, 2 and 1.2, respectively, at 72 vs. 1 hpi. In contrast, only serially centrifuged GII.1 showed consistently successful replication. However, using newer HIE passages and the MagMAX kit resulted in mean log fold increases for serially centrifuged GII.1, GII.4 and GII.6 (1.6, 2.3 and 1.8 log, respectively) that were similar to serially filtered samples. Therefore, HuNoV fecal sample processing and HIE age can affect virus replication in the HIE model.

## 1. Introduction

Human noroviruses (HuNoVs) are small (28–35 nm in diameter), non-enveloped, single-stranded RNA viruses that belong to the genus *Norovirus* within the *Caliciviridea* family. The genus *Norovirus* is classified into ten genogroups that are further divided into 49 genotypes (9 GI, 27 GII, 3 GII, 2 GIV, 2 GV, 1 GVII, 1 GVIII, 1 GIX and 1 GX) [1]. However, only GI, GII, GIV, GVIII and GIX infect humans, and the GII.4 HuNoV is the most prevalent genotype worldwide [2]. Globally, HuNoV is the most common etiologic agent of diarrheal disease among all age groups, causing about one-fifth of cases, accounting for an estimated USD 60.3 billion in economic burden [3,4]. The virus is also the most common cause of diarrheal death, causing over 200,000 deaths worldwide [3]. In the United States, HuNoV incidence among medically attended acute gastroenteritis cases is the highest in children under the age of 5 years and in adults above the age of 65 years [5]. Human norovirus is transmitted through the fecal–oral route, and infections occur by ingesting contaminated food or water, or by transmission from contaminated surfaces and through person-to-person contact in closed areas such as hospitals, schools, nursing homes, prisons or cruise ships [6]. In the US, HuNoV is the leading cause of foodborne illnesses, causing an estimated 58% of all cases [7]. This highlights the need to devise control measures to combat HuNoV illnesses and foodborne outbreaks.

Historically, the lack of a reproducible cell culture system for HuNoV hindered research into anti-viral agents and vaccine development as well as effective interventions to control HuNoV outbreaks. Since its discovery in the 1970s, many research groups have attempted to propagate the virus in mammalian cells without success [8,9,10,11,12]. A breakthrough occurred in 2016 when multiple HuNoV genotypes were reported to replicate in a human intestinal enteroids (HIEs) culture model [13]. In 2018, the CDC group reported the successful reproducibility of the HIE infection model for HuNoV [14]. The HIE model is an emerging experimental, diagnostic and therapeutic tool in the medical field [15]. It is based on isolating single intestinal crypts taken from human intestinal biopsies that are then grown into multicellular self-organized, non-transformed, 3D cultures referred to as epithelial mini-guts [15]. These mini-guts contain stem cells and all intestinal epithelial cells grown inside a laminin- and collagen-rich 3D Matrigel [15,16]. The intestinal epithelial cells that make up these mini-guts are comprised of absorptive (enterocytes) and secretory (goblet, enteroendocrine and Paneth) cells [15]. Inside each mini-gut, enterocytes form the brush border surrounding a single luminal compartment which receives secretions from the other cells [15]. Thus, the HIE model closely resembles the architecture, biology and physiology of the epithelium of the human small intestine [15,16,17].

The details of the initial procedure for maintenance, passage, monolayer differentiation and HuNoV infection of HIE were previously published [18]. Briefly, for HuNoV, the HIE cultures are passaged by first disrupting the Matrigel, and mechanically dissociating the larger multicellular structures into smaller structures and then diluting them 1:2 in new Martigel [16,18]. Mechanically disrupted HIE rapidly reseal and continue to grow for another week before being passaged again [16]. For establishing HIE monolayers, the 3D HIE cultures are dissociated into single cells using trypsin, vigorous pipetting and passing through a 40 µm cell strainer; then, the cells are counted and plated on duplicate collagen-coated 96-well plates [18]. The HIE monolayers are then differentiated within 24 h for the next 5 days before being susceptible to infection with HuNoV [14,18].

Human norovirus was shown to infect enterocytes in the differentiated HIE monolayers [13]. Although cytopathic effects (CPEs) such as cell rounding and monolayer destruction can sometimes be observed in the HIE model, the routine basis of determining the replication success in the HIE infection model is based on the quantification of an increase in virus RNA titer using reverse transcription quantitative polymerase chain reaction (RT-qPCR). It was shown that, at 1 h post-infection (hpi), HuNoV attaches to the cells of the monolayers with no replication happening for the first 6 hpi; however, viral polyprotein synthesis and processing can be detected at 12 hpi [13]. Replication then peaks at 24 hpi and plateaus till at least 96 hpi [13]. Therefore, the viral RNA titers at the 1 hpi constitute the baseline of viruses that attach to the cells. Hence, in routine HuNoV infections of HIE, the viral inocula are removed at 1 hpi and the monolayers are subjected to 2–3 washings before being frozen at −80 °C. Another duplicate plate is incubated further for at least 24 hpi, before being frozen. The detection of at least a 3-fold increase (~0.5 log fold increase) in viral RNA titer at 24, 72 or 96 hpi in comparison to the virus RNA titer at 1 hpi indicates the successful replication of HuNoV [19].

Several factors may affect the successful replication of HuNoV in the HIE monolayers. Successful replication of HuNoV in HIE was initially reported for only four genotypes, GII.4, GII.3, GII.17 and GI.1, in human jejunal enteroid monolayers [13]. A subsequent study reported the successful replication of 6 GII genotypes (GII.1, GII.2, GII.3, GII.14 and GII.17 and multiple GII.4 variants), but not GI, GIV or other GII genotypes, including GII.6 [14]. Another study reported that the use of HIE commercial culture media (Intesticult from Stem Cell Technologies) during propagation, plating and differentiation enhanced the replication of HuNoV strains that replicated poorly using in-house made media [19]. The strains that were reported to show successful replication included one GI.1 and 11 GII genotypes (GII.2, GII.3, GII.4, GII.6, GII.7, GII.8, GII.12, GII.13, GII.14 and GII.17), but not GI.3 or 8 GII.4 strains [19]. Thus, within the same genotype, such as GII.6 and GII.4, some variants may or may not show replication in HIE, depending on HIE culture conditions [14,19]. Factors such as bile and appropriate histoblood group antigen expression on HIE were found to be critical or to enhance replication of certain HuNoV genotypes in HIE, while other factors such as the addition of trypsin and pancreatin failed to enhance HuNoV replication [13]. Other factors that were previously tested for their effect on HuNoV replication in HIE models included HIE age (i.e., passage number which reflects age in weeks of maintaining the HIE in culture) and the volume of HuNoV inoculum (100, 200, 250 or 300 µL) used for infecting the HIE [20]. It was found that older passages at 40–49 resulted in 2% odds of detecting infectious HuNoV GII.4 Sydney as compared to newer passage numbers at 20–29 [20]. In addition, using 200 µL to infect HIE monolayers had reduced odds of detecting infectious HuNoV as compared to using 100 µL [20]. This suggests that further optimization of factors affecting HIE and/or the fecal samples to achieve replication of HuNoV belonging to multiple or within the same genotype is needed.

Fecal sample processing has received little attention for its effect on HuNoV replication in HIE. This is because HuNoV fecal samples are traditionally prepared by serial filtration, i.e., by making fecal suspension in phosphate-buffered saline (PBS) followed by a centrifugation step (≤3000× *g* for ≤30 min) then followed by filtration through a series of filters with a decreasing pore sizes to remove fecal particles [13]. One study compared one fecal sample (positive for HuNoV GII.4) processed by filtration through 0.45 µm filters followed by chemical treatment with Vertrel XF or followed by semi-purification on sucrose cushion using ultracentrifugation (95,000× *g* for 3 h); the authors reported no significant differences on HuNoV replication in HIE among the three fecal processing methods [20]. Another recently reported fecal sample processing method relies on enriching the final sample with virus-containing vesicles. This method is based on successive centrifugation steps with increasing speeds and is referred to as “Serial centrifugation”. A recent study found that a pooled fecal sample obtained from three HuNoV infected patients (presumably with a GII genogroup) processed by serial centrifugation enrich for vesicles containing HuNoV [17]. Furthermore, purified HuNoV-vesicles were found to be infectious in the HIE model, showing ~2 log fold increase at 96 hpi in comparison to 1 hpi [17]. However, the authors did not mention the specific genotype(s) in those pooled fecal samples, nor did they do a direct comparison of serial centrifugation (without further laborious steps to isolate vesicles) to the traditional serial filtration method to determine whether there is an effect on HuNoV replication in HIE. Therefore, the objective of this manuscript was to investigate whether serially centrifugated fecal samples affect replication of HuNoV in the HIE model. The effect of RNA extraction kit (QIAmp versus MagMAX) and HIE age (newer versus older) on serially centrifuged HuNoV fecal samples replication in HIE were also investigated.

## 2. Materials and Methods

### 2.1. HIE Maintenance and Propagation

Frozen vials of J2 jejunal HIE cells (passage 9) were received from Dr. Mary Estes’s laboratory at Baylor College of Medicine (Houston, TX, USA) and were stored in liquid nitrogen until use. Three-dimensional in vitro cultures of HIE were maintained and propagated as described previously [14,18]. Briefly, the frozen HIE vial was thawed and transferred into 10 mL ice-cold complete media without growth factors (CMGF-) made of Advanced DMEM/F-12 supplemented with 1% of each of 1 M HEPES, penicillin–streptomycin (10,000 U/mL) and GlutaMax^TM^ supplement (Thermo Fisher Scientific, Waltham, MA, USA). The resuspended HIE cells were centrifuged at 100× *g* for 3 min at 4 °C. Then, Corning^TM^ Matrigel™ GFR membrane (Fisher Scientific, Waltham, MA, USA) was added to the pellet, mixed and aliquoted as droplets of 30 µL per well of a Nunc^TM^ cell culture treated 24-well plate (Fisher Scientific). The plates were left at room temperature (RT) for a minute to solidify the Matrigel, and then incubated upside down at 37 °C for an additional 5 min. Then, as shown in a previous HIE optimization study [19], each HIE dome received 500 μL of IntestiCult^TM^ (INT) human growth media made up of equal volumes of human basal media and organoid supplement organoid growth medium (Stemcell technologies, Cambridge, MA, USA) supplemented with 0.02% 5 mM Y-27632 dihydrochloride (Sigma-Aldrich, Burlington, MA, USA). This media for proliferation of HIE was referred to as INTp and was changed every other day for 7 days. After that the 3D cultures were passaged as 1:2 to maintain their growth for 4 weeks. Passaging was performed by disrupting the Matrigel using ice-cold CMGF- and pipetting the contents of the well up and down. Then, using a 1 mL syringe with a size 25G × 0.62 in. needles (Fisher Scientific), the contents of each well were aspirated 2–3 times before being centrifuged at 100× *g* for 3 min at 4 °C. The pellet was then re-suspended in fresh Matrigel and transferred into the desired number of wells. Following 4 weeks of passage, the expanded 3D HIE cultures were used to establish monolayers for HuNoV infections.

### 2.2. Establishing and Differentiation of HIE Monolayers

Five-week-passaged HIE cultures were used to prepare HIE monolayers, as described previously [14,18]. Briefly, the HIE cells were collected from multiple wells using ice-cold 0.5 mM EDTA in DPBS (Thermo Fisher Scientific). The HIE cells were then pelleted by centrifugation at 200× *g* for 5 min at 4 °C. Then, the pellet was dissociated by adding 0.05% trypsin/0.5 mM EDTA and incubating at 37 °C for 4 min, followed by the addition of CMGF- containing 10% FBS to inactivate trypsin. The dissociated pellet was passed through a 40 µm Falcon^TM^ cell strainer (Fisher Scientific) and then pelleted by centrifugation at 400× *g* for 5 min at RT. The pelleted HIE cells were re-suspended in IntestiCult + Y. The cell count was obtained by mixing 10 μL of cells with 10 μL of 0.4% trypan blue solution in PBS (VWR, Radnor, PA, USA) and using the Countess™ II automatic cell counter. A solution of collagen IV (stock 33 μg/mL in 0.6% acetic acid) from human placenta (Sigma-Aldrich) was diluted 1:30 (*v*/*v*) in sterile water and used to coat the desired number of wells of Fisherbrand^TM^ 96-well cell culture treated plates (Fisher Scientific). The plates were incubated for at least 2 h at 37 °C, followed by removal of excess collagen liquid. The HIE cells were diluted to 1 × 10^6^ viable cells/mL using INTp and plated on the pre-coated collagen plates (100 µL/well). The plates were then incubated at 37 °C for 24 h. Then, the INTp was replaced with differentiation medium consisting of equal volumes of CMGF- and human basal media (referred to as INTd), as reported in recent studies [19,21]. The cell monolayers were differentiated for 4 days, while replacing the differentiation media every 48 h. When the monolayers reached 90% confluency (usually within 5 days), they were used to test infectivity of HuNoV fecal samples.

### 2.3. HuNoV Infection of Differentiated HIE Monolayers

Infection of HIE monolayers with HuNoV-positive fecal samples (prepared as described below) was performed as described previously [14]. Briefly, the differentiated HIE monolayers were washed once using ice-cold CMGF- followed by the addition of HuNoV diluted in infection media consisting of CMGF- supplemented with 500 µM glycochenodeoxycholic acid (GDCA) (Thomas Scientific, Swedesboro, NJ, USA) and 50 µM ceramide (Santa Cruz Biotechnology, Dallas, TX, USA). Triplicate wells of differentiated HIE wells were inoculated with 100 µL of HuNoV in duplicate plates intended for the 1 and the 72 hpi. The plates were incubated at 37 °C. Following the 1 hpi, both plates were washed 3 times using 200 µL CMGF- to remove the unbound HuNoV. Then, 100 µL of INTd supplemented with 500 µM GDCA and 50 µM ceramide was added per well and the 1 h plate was immediately frozen at −80 °C. The duplicate plate was frozen after an additional 72 hpi at 37 °C. A negative control was included in each plate consisting of HIE monolayers treated similar to infected wells but without the addition of HuNoV.

### 2.4. Fecal Processing by Serial Centrifugation and Serial Filtration

Limited quantities of HuNoV fecal samples belonging to GII.1 [22], GII.4 [21] and GII.6 [23] genotypes that were known to replicate in HIE were received courtesy of Dr. Samantha Wales (FDA/CFSAN). Fecal suspensions (10%) were prepared in 1X PBS and these were processed by either serial centrifugation [17] or serial filtration [13]. Briefly, 10% fecal suspensions were vortexed for at least 1 min to mix the viruses thoroughly. For serial centrifugation, the fecal suspensions were then centrifuged at 500, 1500, 2500, 3500 and 5000× *g* at 4 °C for 5 min per centrifugation speed. For serial filtration, the fecal suspensions were filtered using sterile 1.2 μm, then 0.8 µm cellulose acetate filters (VWR International, Radnor, PA, USA), then 0.45 µm followed by 0.22 μm low-protein binding Millex^®^polyethersulfone filters (MilliporeSigma, Burlington, MA, USA). After each filtration and centrifugation step, the filtrates and the supernatants, respectively, were vortexed at high speed for 1 min. The final supernatants or filtrates were aliquoted into 50 µL aliquots and stored at −80 °C for later testing on HIE. Aliquots were used a maximum of two times to avoid repeated freezing and thawing, as mentioned previously [19]. For comparing fecal processing methods, the QIAamp^®^ Viral RNA Mini kit (QIAGEN, Germantown, MD, USA) was used for RNA extraction from HIE well, as described below.

### 2.5. RNA Extraction Using QIAamp and MagMAX Kits

Because serially centrifuged samples did not show successful replication in HIE for all genotypes tested, we first explored the effect of RNA extraction on RNA titers obtained from HIE at 1 and 72 hpi. Using serially centrifuged fecal samples only, the manual RNA extraction QIAamp kit was compared to the MagMAX™ *mir*Vana™ Total RNA Isolation Kit (Thermo Fisher Scientific), which is designed to be run on a semi-automated King Fisher Duo Prime machine (Thermo Fisher Scientific). These kits were used to extract RNA from the 1 and 72 hpi HIE wells following manufacturers’ instructions. Briefly, for the QIAamp kit, 160 µL of the prepared AVL lysis buffer with carrier RNA was added to the HIE wells. The contents of the treated monolayers were then transferred to 1.5 mL tubes. The samples were then vortexed for 15 s and incubated at RT for 10 min. Following that, 560 µL of pure ethanol (≥95%) was added to the tubes and vortexed for 15 s. The samples were transferred to a QIAamp Mini Spin column and centrifuged at 6000× *g* for 1 min. Two subsequent washes with AW1 and AW2 buffers were followed with a final step to elute RNA using a 60 μL of AVE buffer. For the MagMAX kit, the lysis binding mix, TURBO DNase solution and binding beads mix were first prepared according to manufacturers’ instructions. Then, 200 µL of the lysis binding mix was added to the HIE wells and incubated at RT for 5 min, before transferring to row H of the King Fisher (Thermo Fisher Scientific) 96-well, deep-well plate. Then, 20 µL of the binding beads mix was added to each sample and the rest of the KingFisher plate was prepared according to manufacturer instructions. At the end of the extraction protocol, the recovered RNA samples (60 µL) were transferred from the KingFisher plate to sterile 1.5 mL MaxyClear snaplock microcentrifuge tubes (Thomas Scientific).

### 2.6. Two Intervals of HIE Passages

Next, we explored the effect of HIE age on the replication of serially centrifuged HuNoV in HIE. Two HIE passages referred to as “newer” (passage 5 to 19) and “older” (passage number 20–34) that were 15 weeks apart in cell culture were compared for their ability to show successful replication of serially centrifuged HuNoV fecal samples. Fecal samples for GII.1, GII.4 and GII.6 were prepared by serial centrifugation as described above. The HIE monolayers made from the two HIE age batches were infected with HuNoV as described above. The control- and infected-HIE were extracted using the MagMAX kit.

### 2.7. HuNoV-Specific RT-qPCR

The RT-qPCR was performed using the TaqPath™ 1-Step RT-qPCR Master Mix (Fisher Scientific). This Master Mix consists of Fast DNA polymerase, thermostable MMLV enzyme, Uracil-N glycosylase, DNTPs including dUTP, RNase inhibitor, ROX TM dye (passive reference) and buffer components optimized for maximum sensitivity and tolerance to several common RT-qPCR inhibitors. The sequence of the primer and probes for HuNoV GII were used as described previously [24]. All reactions were performed by taking 5 µL of each RNA sample and mixing it with 1X Master Mix, 200 nM of each of the forward (COG2F) and reverse (COG2R) primers in addition to 100 nM of the GII HuNoV RING2 probe. The final volume of the PCR reaction was 20 µL. A standard curve was prepared by tenfold serial dilution of the synthetic HuNoV GII (ATCC, Gaithersburg, MD, USA) starting from 5 × 10^5^ to 0.5 GE/µL. The amplification cycling conditions were reverse transcription step at 50 °C (15 min), then one step at 95 °C (5 min), followed by 45 cycles of 95 °C (15 s) and 60 °C (35 s). The prepared PCR plates were run on the QuantStudio 5 machine and analyzed by the QuantStudio™ Design & Analysis Software V3.0.1. Each RT-qPCR run had control positives and negatives. The positive control was a known GII.4 HuNoV sample that was extracted with every RNA extraction run. The negative control was 15 µL of the master mix with 5 µL of PCR grade water. Each RNA sample was tested in duplicate.

### 2.8. Statistics

The GraphPad Prism software version 5 (GraphPad Software, San Diego, CA, USA) was used for all statistical analyses. The fold increase was calculated by dividing the 72 hpi RNA titers (GE/well) by the 1 hpi. Then, the entire dataset for RNA titers and fold increases was transformed to log_10_. As previously published, successfully replication was defined as 3-fold increase (i.e., 0.5 log fold increase) at the 72 hpi [19]. The unpaired student *t*-test for comparison of two means and one-way analysis of variance (ANOVA) followed by Tukey for comparison of multiple means were used to determine significant differences in means of various treatments or time points. All experiments were repeated 3 times, and each fecal sample was run in triplicate wells of HIE monolayers for the 1 and 72 hpi, respectively. Then, each RNA sample was run in duplicate for the RT-qPCR assay. Linear regression analyses were performed on Ct values and RNA titers of GII synthetic RNA to determine the slope and Y-intercept of the equation as well as the goodness-of-fit of linear regression (R^2^). Significance was determined when the *p* value was less than 0.05 and are denoted in the figures by different alphabets or by asterisks (* *p* < 0.05, ** *p* < 0.001 and *** *p* < 0.0001). Data are expressed as the mean ± standard error (SE).

## 3. Results

### 3.1. RT-qPCR Limit of Detection for HuNoV GII RNA

**Figure 1:**
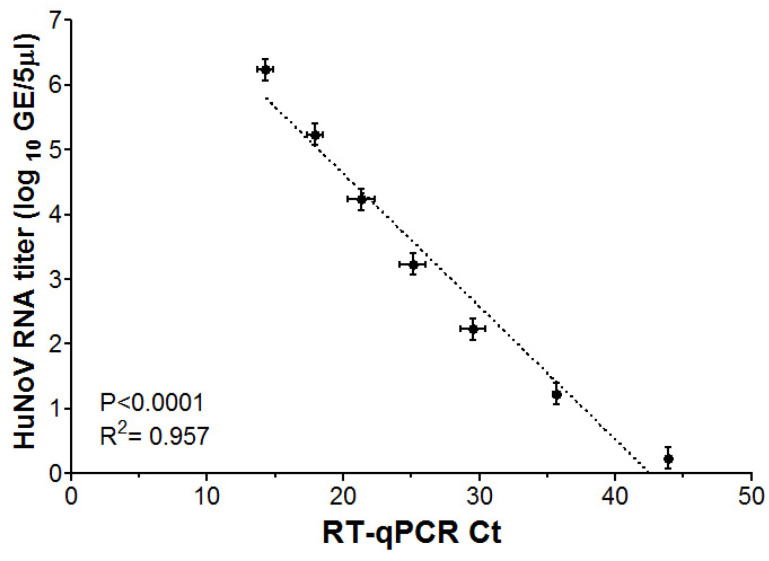
Scatter plot between RT-qPCR Ct values and HuNoV RNA titers (log GE/5 µL). The ATCC (VR-3235SD^TM^) synthetic norovirus GII RNA was serially diluted tenfold before running on the RT-qPCR assay. Error bars represent standard errors around the mean. Dashed line indicates the best-fit line from linear regression analysis.

The HIE culture method for HuNoV is based on viral RNA quantification from infected HIE wells using RT-qPCR. The limit of detection (LOD) is used to determine the lowest virus RNA level that can be quantified by RT-qPCR. The RT-qPCR Ct values and the log RNA titers for HuNoV GII RNA (ATCC) showed a significant linear relationship with R^2^ = 0.957 (Figure 1). The lowest HuNoV GII viral RNA that can be detected with this RT-qPCR was found to be ~0.3 log GE/5 µL, corresponding to 10 viral particles per HIE well or 1 log GE/well. Therefore, for subsequent experiments, HuNoV RNA extracted from HIE wells that were undetectable in the RT-qPCR assay were assigned half of the value of the detection limit, i.e., 0.5 log GE/well.

### 3.2. Effect of Fecal Sample Processing on HuNoV Replication in HIE

HuNoVRNA titers from fecal samples used in HIE infections were not significantly different among the three genotypes or between the two fecal sample processing methods, i.e., serial centrifugation or serial filtration (Table 1).

**Figure 2:**
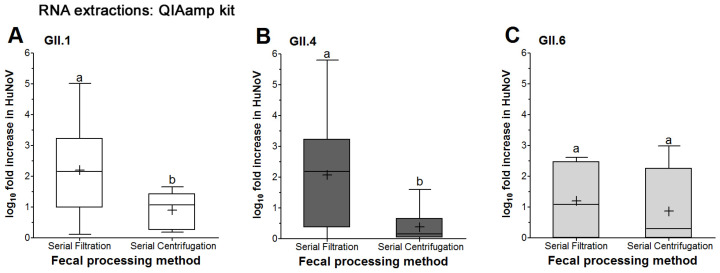
HuNoV replication in HIE as affected by fecal sample processing. Fecal samples for (**A**) GII.1, (**B**) GII.4 and (**C**) GII.4 were processed by either serial filtration or serial centrifugation. RNA extractions from HIE wells were performed using the QIAamp^®^ Viral RNA Mini kit followed by RT-qPCR to quantify the viral RNA titers at 1 and 72 hpi. Boxes show the 25th percentile, median, 75th percentile and mean (+), while whiskers show the maximum and minimum values for log fold increases in HuNoV RNA titers in HIE at 72 compared to 1 hpi. Experiments were repeated 3 times, and each fecal sample was run in triplicate wells of HIE monolayers for the 1 and 72 hpi, respectively. Different letters indicate significant differences between means (*p* < 0.05).

Both fecal processing methods showed successful replication for GII.1 in HIE, as shown by significant increase in the 72 vs. 1 hpi RNA titers (Table 2). Serially filtered GII.1 showed a maximum fold increase of 5 log vs. 1.6 log for serially centrifuged GII.1 (Figure 2A). The mean log fold increase for serially filtered GII.1 was significantly higher than that of serially centrifuged samples (2.2 vs. 0.9 log, respectively) (Figure 2A). Taken together, GII.1 showed replication in HIE under both fecal processing methods.

For GII.4 infection of HIE, serially filtered samples showed significant increase in RNA titers at 72 vs. 1 hpi, while serially centrifuged samples did not (Table 2). Serially filtered GII.4 showed a maximum log fold increase of 5.8 log vs. 1.6 log for serially centrifuged GII.4 (Figure 2B). The mean log fold increase for serially filtered GII.4 was significantly higher than that of serially centrifuged samples (2 vs. 0.4 log, respectively) (Figure 2B). Taken together, serially centrifuged GII.4 did not show consistent successful replication in HIE.

For GII.6 infection in HIE, similar to the GII.4, the serially filtered samples showed significant increase in RNA titers at 72 vs. 1 hpi, while the serially centrifuged samples did not (Table 2). The log fold increase data for serially filtered GII.6 showed a maximum log fold increase at 2.6 log vs. 3 log, for serially centrifuged GII.6 (Figure 2C); however, there were no significant differences in the mean log fold increase between the two fecal processing methods (1.2 vs. 0.9 log, respectively) (Figure 2C). Taken together, this indicates that serially centrifuged GII.6 did not show consistent successful replication in HIE.

### 3.3. Effect of RNA Extraction Kit on Serially Centrifuged HuNoV Replication in HIE

Next, to explore whether serially centrifuged HuNoV fecal samples infection in HIE can be optimized, we first investigated the effect of RNA extraction kit type and then the effect of HIE age on replication, i.e., log fold increase in HuNoV in HIE. Infections with the serially centrifuged fecal samples for HuNoV GII.1, GII.4 and GII.6 were repeated in HIE, but the HIE monolayers were extracted using the MagMAX semi-automated kit. This was compared to the above results of HIE infections with serially centrifuged samples that were extracted using the QIAamp kit.

**Figure 3:**
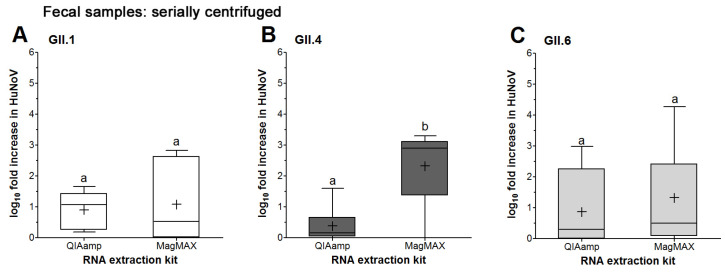
HuNoV replication in HIE as affected by RNA extraction kit. Fecal samples for (**A**) GII.1, (**B**) GII.4 and (**C**) GII.6 were processed by serial centrifugation. RNA extractions from HIE wells were performed using the QIAamp^®^ Viral RNA Mini kit or MagMAX™ *mir*Vana™ Total RNA Isolation kit followed by RT-qPCR to quantify the viral RNA titers at 1 and 72 hpi. Boxes show the 25th percentile, median, 75th percentile and mean (+), while whiskers show the maximum and minimum values for log fold increases in HuNoV RNA titers in HIE at 72 compared to 1 hpi. Experiments were repeated 3 times, and each fecal sample was run in triplicate wells of HIE monolayers for the 1 and 72 hpi, respectively. Different letters indicate significant difference between means (*p* < 0.05).

Using the MagMAX kit, serially centrifuged GII.1 did not show significant increases in RNA titers at the 72 vs. 1 hpi this time, while serially centrifuged GII.4 was improved (in comparison to QIAamp results) and did show significant increases (Table 2). Serially centrifuged GII.6 again did not show significant increases in RNA titers at the 72 vs. 1 hpi (Table 2). The mean log fold increase using the MagMAX kit for GII.1 and GII.6 were not significantly different from those using the QIAamp kit (Figure 3A,C). However, there was a significant enhancement in the GII.4 mean log increase using the MagMAX kit as compared to the QIAamp kit (1.7 vs. 0.4 log, respectively) (Figure 3B). Whiskers show that the maximum log fold increases for GII.1, GII.4 and GII.6 were obtained using the MagMAX kit at 2.8, 3.3 and 4.3 log (Figure 3A–C), respectively. Taken together, serially centrifuged GII.4 infections in HIE that were extracted using MagMAX showed significant 72 to 1 hpi increase and an enhancement in log fold increases as compared to those extracted using QIAamp (above the 0.5 cutoff), and thus were considered to successfully replicate in HIE. In contrast, GII.1 with QIAamp extractions showed a significant increase in 72 vs. 1 hpi RNA titers, but with MagMAX, the difference was not significant, even though it was above the 0.5 log cutoff. Therefore, factors other than RNA extraction kit type were affecting the consistent replication of these serially centrifuged HuNoV genotypes in HIE.

### 3.4. Effect of HIE Age on Serially Centrifuged HuNoV Replication in HIE

All the trials performed above were performed on HIE passages ranging from 7 to 28 (Table 2). To investigate whether the HIE continuous passaging period “age” in cell culture affected serially centrifuged HuNoV replication in HIE; two HIE batches were maintained at 15 weeks apart. Newer HIEs were defined from passage numbers 5 to 19 and older HIEs were defined from passage numbers 20 to 34.

**Figure 4:**
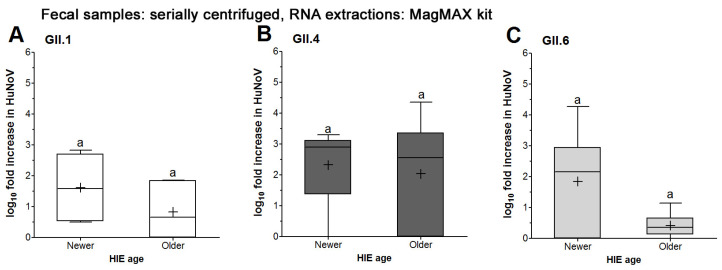
HuNoV replication in HIE as affected by HIE age. Fecal samples for (**A**) GII.1, (**B**) GII.4 and (**C**) GII.6 were processed by serial centrifugation. Two sets of HIE passages were maintained (newer 5–19 and older 20–34) that were 15 weeks apart. RNA extractions from HIE wells were performed using the MagMAX™ *mir*Vana™ Total RNA Isolation kit followed by RT-qPCR to quantify the viral RNA titers at 1 and 72 hpi. Boxes show the 25th percentile, median, 75th percentile and mean (+), while whiskers show the maximum and minimum values for log fold increases in HuNoV RNA titers in HIE at 72 compared to 1 hpi. Experiments were repeated 3 times, and each fecal sample was run in triplicate wells of HIE monolayers for the 1 and 72 hpi, respectively. Different letters indicate significant difference between means (*p* < 0.05).

In older HIEs, serially centrifuged GII.1 and GII.6 did not show significant increases in RNA titers at the 72 vs. 1 hpi, whereas for newer HIEs, they did (Table 2). The mean log fold increases for GII.1 and GII.6 were consistently higher when infection happened in newer HIEs, as compared to older HIEs. However, this difference was not statistically significant (Figure 4A,C). For GII.4, both older and newer HIEs showed significant increases for the RNA titers at the 72 vs. 1 hpi (Table 2) and the mean log increase was not significantly different between both HIE ages (Figure 4B). Taken together, HIE age affected the successful replication of serially centrifuged GII.1 and GII.6 but not that of serially centrifuged GII.4. It is noteworthy that using newer HIE showed significant increases of 72 vs. 1 hpi RNA titers for the first time, above the 0.5 cutoff, for all serially centrifuged genotypes tested (Table 2).

### 3.5. Overall Comparisons of HuNoV Genotypes among Various Treatments

In general, the lowest 1 hpi RNA titers for GII.1, GII.4 and GII.6 were obtained with serially filtered fecal samples using the QIAamp kit (1.5, 1.8 and 0.6 log GE/well, respectively) which were not significantly different from those obtained with the serially centrifuged fecal samples using MagMAX kit and newer HIEs (2, 0.6 and 0.7 log GE/well, respectively) (Table 2). In general, the highest 1 hpi RNA titers for GII.1, GII.4 and GII.6 were obtained with the serially centrifuged samples using the QIAamp kit, but this treatment also showed the highest 72 hpi titers (Table 2). Using both the MagMAX kit and the newer HIEs gave, in general, significantly lower 1 and 72 hpi RNA titers (Table 2) and higher mean log fold increases (Table 3). This suggested that the 1 hpi RNA titers affect fold increases and outcome of replication.

The overall comparison of log fold increases among the various treatments revealed that using newer HIE and the MagMAX kit resulted in similar mean log increases between serially centrifuged and serially filtered fecal samples for GII.1, GII.4 and GII.6 (1.6, 2.3, 1.8 vs. 2.2, 2 and 1.2 log, respectively) (Table 3).

**Figure 5:**
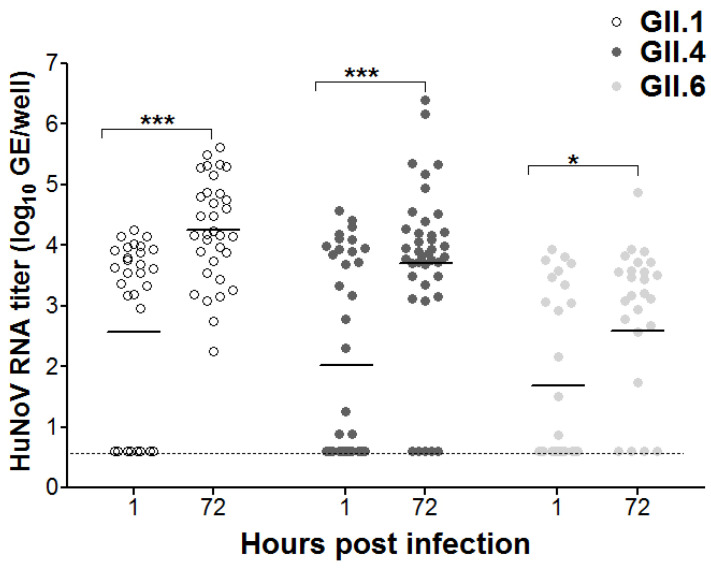
Scatter dot plot showing overall combined data for HuNoV replication in HIE regardless of the fecal processing method, RNA extraction kit or HIE age. Solid lines indicate the means. The dashed line indicates samples that were undetectable by RT-qPCR which were assigned half of limit of detection (i.e., 0.5 log GE/well). Significant differences between 1 and 72 hpi were designated with asterisks.

Finally, data from all the above experiments were analyzed for significant differences between 1 vs. 72 hpi for GII.1, GII.4 and GII.6, regardless of the fecal processing method, RNA extraction kit or HIE age used. For GII.1, GII.4 and GII.6, there were significant increases in the overall mean RNA titers at the 72 vs. 1 hpi, resulting in mean log fold increases of 1.7, 1.7 and 0.9 log, respectively (Figure 5).

## 4. Discussion

Most of the previous HuNoV-HIE studies reported the use of 10% fecal filtrates that were prepared by serial filtration [13,14,19,20]. Traditionally, to recover viruses from fecal samples, the fecal suspensions (in PBS) are centrifuged at 1500–3000× *g* for 10 min at 4 °C once or twice, and the resulting supernatant is passed serially through all or some of the following filters: 5 μm, 1.2 µm, 0.8 µm, 0.45 µm and 0.22 µm filters (what we referred to as serial filtration). A slight modification was followed by the CDC’s HuNoV-HIE manuscript where the fecal solids were removed by one high-speed centrifugation at 10,000× *g* for 10 min before serially filtering the supernatants through 5 μm, 1 μm, 0.45 μm and 0.22 μm filters [14]. One study reported the use of 1% fecal filtrates that were filtered once through 0.22 µm [25]. This variation in fecal sample processing using serial filtration and different filter pore sizes depends on the stool texture [19]. However, the only study that considered the effect of fecal sample processing method on replication of HuNoV in HIE used 10% fecal suspensions in PBS that were filtered through a 0.45 µm filter and then treated with one of the following: Vertrel XF alone; Vertrel XF with subsequent filtration through a 0.45 µm filter; the latter with a subsequent ultracentrifugation step at 95,000 rpm for 3 h on a 20% sucrose cushion to generate more purified the viral particles [20]. The latter study found no association between fold increase in HuNoV GII.4 and any of these processing methods [20]. We followed a different approach for fecal processing which was based on a recent study that showed that serial centrifugation of fecal samples retains viral clusters of HuNoV cloaked inside vesicles [17]. However, in that study, the authors used a pooled fecal sample from three patients, without determining the specific HuNoV genotype(s) used; also, following the serial centrifugation steps, further laborious purifications steps were performed to isolate HuNoV-vesicles [17]. The purified HuNoV-vesicles showed ~2 log fold increase after 96 hpi in HIE [17]. In our study, we showed that, at 72 hpi and without the need of further purifications of vesicles from serially centrifuged samples for GII.1, GII.4 and GII.6, successful replication occurred in HIE with a 1.6, 2.3 and 1.8 log fold increase after implementing simple optimizations such as the use of newer HIE passages and a semi-automated RNA extraction kit. Serially centrifuged samples resulted in comparable log fold increases to serially filtered samples for three HuNoV genotypes. The advantage of serial centrifugation is that it offers simpler fecal sample preparation without the need of multiple filters which usually clog and lead to sample loss.

Total RNA is usually extracted from each HIE well including the cells and the supernatants. Previous HuNoV-HIE studies differed in the method of RNA extraction used. For example, a Ribozol kit was used in the original HuNoV-HIE publication [13] and in another recent study [26], while the MagMAX Total RNA Isolation Kit with the semi-automated King Fisher machine was used in the CDC publication [14] and two other studies [13,27], and the Direct-zol RNA miniprep kit was used in two other studies [20,28]. In addition, previous studies on HuNoV recovery from food and environmental samples have used the QIAamp kit from QIAGEN for virus RNA extraction [29,30,31]. Furthermore, the QIAamp kit was found to provide the best virus RNA recovery efficiency when compared to five other extraction methods [30]. Therefore, the manual QIAamp kit was compared to the most used MagMAX kit, which is designed to run in a semi-automatic machine. In general, the QIAamp viral RNA kit is not specifically designed for nucleic acid extractions from cells and is based on adsorbing nucleic acids to silica membranes in columns, followed by washing off contaminants which may inhibit downstream RT-qPCR. Furthermore, a carrier RNA is initially added to each sample to enhance binding of nucleic acids to the membranes and to further prevent their degradation from RNases. In contrast, the MagMAX kit is designed to extract viral RNA from cells and is based on adsorbing total nucleic acids to magnetic bead, followed by washing and then treatment with DNases. Thus, the MagMAX kit results in purified RNA while the QIAamp kit recovers both RNA, DNA and carrier RNA. Furthermore, the manufacturers of TaqPath^TM^ Master Mix recommend using RNA concentrations at ~100 ng/reaction of RT-qPCR. Because the QIAamp kit lacks a genomic DNA removal step while the MagMAX kit includes a genomic DNA removal step, the QIAamp kit generates much higher nucleic acid yields from extracted HIE wells than the MagMAX kit (an average of 100 µg/µL vs. 10 µg/µL, respectively). However, this difference in nucleic acid yield was not an issue because the QIAamp kit, when used to extract HIE wells inoculated with serially filtered samples, showed successful replications for all tested genotypes, but only showed successful replication for GII.1 serially centrifuged fecal samples. The use of the MagMAX kit showed significantly higher 72 vs. 1 hpi RNA titers and mean log fold increase only for serially centrifuged GII.4 in comparison to serially centrifuged GII.4 extracted using the QIAamp kit. In contrast, the opposite result was observed for GII.1, whereby the QIAmp kit showed significant 72 vs. 1 hpi increase for serially centrifuged GII.1, while MagMAX did not. Therefore, successful replication is independent of the kit type tested; however, for serially centrifuged HuNoV fecal samples, other factors, such as HIE age discussed below, may have played a larger role in affecting their replication.

In addition to variation in RNA extraction methods, the final volume of the RT-qPCR reaction is different among various HuNoV-HIE studies. For example, some used 15 µL [13], while others did not report their measure [14,20,27]. We used a final volume of 20 µL as recommended by the manufacturer of the TaqPath^TM^ 1-step RT-qPCR Master Mix. In addition, these studies used 40 cycles in the RT-qPCR protocol; meanwhile, others did not report the number of cycles used. We used 45 cycles in the RT-qPCR protocol. Also, studies differed in their type of synthetic HuNoV transcript used for the generation of the standard curve. For example, in-house-made recombinant HuNoV RNA transcripts for various HuNoV genotypes were used to calculate the viral genome equivalents in RNA samples [13,14,20]. These in-house-made standard curves cannot be reproduced by other labs. We used a commercially available HuNoV RNA for GII of known RNA copy number per µL. All of these factors may have contributed to the lower RT-qPCR detection limit reported in our study (10 GE/well) versus what was reported previously: 4000 GE/well [14], 1200 GE/well [13], 886 GE/well [20] and 110 GE/well [25].

The titer of the virus inocula was shown in previous studies to inconsistently affect HuNoV replication in HIE [14,19,20,25,32]. For example, some HuNoV fecal samples that showed successful replication had a significantly higher titer than those that did not, while others with high titers did not show any replication [14,19,20]. Studies suggest that there is no one ideal input titer for successful replication of HuNoV; however, successful replication is more likely with higher virus titer [19,20]. In addition, there is a minimum required, titer below which no replication is expected to occur. For example, the minimum input viral RNA titer required for successful infection of half of the HIE wells (infectious dose 50%) was determined to be 1.2 × 10^3^ and 2 × 10^4^ genome equivalents (GE)/well for GII.4/2012 and GII.3, respectively, after 7 days post-HIE infection (i.e., 3 and 4.3 log GE/well) [13]. However, the limit was different for different strains of GII.4 such as GII.4 Sydney (4.4 × 10^2^ GE/well), GII.4 Den Haag (2.1 × 10^3^ GE/well) and for GII.3 (4 × 10^3^ GE/well) after 3 days post-HIE infection (i.e., 2.6, 3.3 and 3.6 log GE/well, respectively) [14] and for another strain of GII.4 Sydney as 1.4 × 10^3^ GE/well [20]. Therefore, we infected HIE with RNA titers above the minimal infectious dose (~4.3 log GE/well estimated from the previous studies) to eliminate this factor as a reason for failed replication.

Most of the previous HIE-HuNoV studies did not mention the optimum HIE passage number used in HuNoV infections [13,19,27,28]. The initial CDC manuscript mentioned the use of HIE passaged continuously for 4 months reaching maximum passage of 25 [14]. A previous study noted that GII.3 only replicated in new HIE without providing specific passage numbers [13]. One study specifically mentioned the use of HIE passages with numbers less than 20 to determine the replication of HuNoV GII.4 fecal samples in HIE [25]. The only study that compared different sets of HIE ages used passages that are 9 weeks apart, starting from 20 to 29, 30 to 39, 40 to 49 and 50 to 59, to reveal a 2% odds of detecting infectious GII.4, only when passages 40–49 were compared to passages 20–29 [20]. In our study, replication of serially centrifuged GII.4 was not affected by HIE age; however, older HIE passages (#20–34) showed no significant increases at 72 vs. 1 hpi for serially centrifuged GII.1 and GII.6 as compared to newer HIE passages (#5–19). In addition, when all treatments were compared, only newer HIE (passages 5–19) showed significant increases in 72 vs. 1 hpi RNA titers for all three serially centrifuged genotypes tested. The latter indicates that HIE age is an important factor to consider in HuNoV replication in HIE. Further studies are needed to explore what physiological changes occur as HIE age changes and their effect on HuNoV replication.

Among the several experiments conducted, it was observed that variation in the titer at 1 hpi affected the outcomes of replication (log fold increase). In previous manuscripts, following the 1 hpi infection, the monolayers were washed twice, which resulted in viral RNA titers of 3–4 log GE/well at the 1 hpi [13]. Similarly, in our study, we often observed that the RNA titers at the 1 hpi ranged between 3 and 4 log GE/well, despite washing the monolayer three times following the 1 hpi period. However, a low titer between 0.5 and 1 log GE/well at the 1 hpi was also detected, especially in serially filtered samples and serially centrifuged samples that were used in the infection of newer HIEs along with RNA extractions using MagMAX. It is not clear what conditions may have contributed to these lower titers at the 1 hpi. A high 1 hpi titer can lead to nonsignificant differences in comparison to the 72 hpi titer, as observed in our study among serially centrifuged samples that were extracted using QIAamp kit (Table 2). Further studies are needed to explore HIE washing buffers or HIE conditions that would allow the removal of excess unbound HuNoV to HIE cells which would result in lower 1 hpi and better log fold increase at 72 hpi.

The cutoff or threshold value by which HuNoV is considered to successfully replicate in HIE varied between studies. From the original research group developing HIE for HuNoV, a 0.5 log increase at 24 hpi vs. 1 hpi was defined as indictive of successful HuNoV replication [19]. Another group reported that, if the fold increase at 72 vs. 1 hpi was less than 5, which is ~0.7 log, samples were considered negative for replicating HuNoV [20]. A third group considered a ≥10-fold increase (i.e., 1 log) at 72 w.r.t 1 hpi to indicate successful viral replication [25]. We applied the threshold of 0.5 log fold increase; however, we sometimes observed a log fold increase >0.5, but there were no significant differences at *p* < 0.05 between viral RNA titers at the 72 and 1 hpi (Table 2 and Table 3). Therefore, the criteria for successful replication should also include standard variation around the 0.5 cutoff and statistical significance between RNA titers at 72 and 1 hpi. This would be very important for food and environmental samples that usually contain low titers of HuNoV. Nevertheless, in our study, the overall mean log fold increase, based on all HIE trials regardless of fecal sample processing, RNA extraction or HIE age, was >0.5 for all tested genotypes; this indicates the presence of infectious viruses in these fecal samples.

## 5. Conclusions

In conclusion, the choice of the fecal sample processing method and HIE age may affect replication outcomes, i.e., the detection of infectious HuNoVs, depending on the HuNoV genotype in question. However, serial centrifugation processing of fecal samples for HuNoV offered a simpler approach with mean log fold increases in HuNoV RNA titers at 72 hpi similar to the traditional serial filtration method when the MagMAX kit and newer HIE age were used.

## Figures and Tables

**Table 1:** Summary of fecal samples positive for HuNoV used in this study. Means with significant differences are indicated by different alphabets: capital letters indicate comparison within a column, i.e., among genotypes, and small letters indicate comparisons across a row, i.e., between the two fecal processing methods.

Genotype	P-Type	GenBank Accession Number	Serial Filtration Titer (Log GE/Well)	Serial Centrifugation Titer (Log GE/Well)	Reference
GII.1	[Pg]	MW854326	4.7 ± 0.03 Aa	4.6 ± 0.5 Aa	[22]
GII.4	[P16]	MN782359	4.8 ± 0.2 Aa	5.0 ± 0.3 Aa	[21]
GII.6	[P7]	KX268709	4.5 ± 0.08 Aa	4.4 ± 0.6 Aa	[23]

**Table 2:** HuNoV GII.1, GII.4 and GII.6 RNA titers (log GE/well) at 1 and 72 hpi in HIE under various treatments. Data are expressed as mean ± standard error (SE). Means with significant differences are indicated by different alphabets: capital letters indicate comparison within a column, i.e., between 1 and 72 hpi within a specific genotype and a treatment, while small letters indicate comparison of means across a row, i.e., all treatments within either 1 or 72 hpi. HIE passages # 5–19 were defined as “Newer” while those numbered 20–34 were defined as “Older”.

Fecal Processing		Serial Filtration	Serial Centrifugation			
RNA Extraction Kit		QIAamp	QIAamp	MagMAX	MagMAX	MagMAX
HIE passages #		7–28	7–28	7–28	5–19	20–34
GII.1 (log GE/well)	1 h	1.5 ± 0.5 Aa	3.9 ± 0.0 Ab	2.8 ± 0.5 Aab	2.0 ± 0.6 Aab	3.7 ± 0.2 Ab
	72 h	3.7 ± 0.3 Ba	4.8 ± 0.2 Bb	3.7 ± 0.1 Aac	3.7 ± 0.2 Bac	4.3 ± 0.2 Ab
GII.4 (log GE/well)	1 h	1.8 ± 0.5 Aa	4.1 ± 0.08 Ab	1.4 ± 0.4 Ac	0.6 ± 0.0 Aac	1.9 ± 0.5 Aac
	72 h	3.9 ± 0.5 Bab	4.5 ± 0.3 Aa	3.1 ± 0.3 Bb	2.9 ± 0.4 Bab	3.9 ± 0.1 Bab
GII.6 (log GE/well	1 h	0.6 ± 0.0 Aa	2.8 ± 0.5 Ab	1.7± 0.5 Aab	0.7 ± 0.1 Aac	2.7 ± 0.4 Ab
	72 h	1.7 ± 0.4 Ba	3.6 ± 0.0 Ab	2.9± 0.5 Aab	2.4 ±0.7 Bab	2.7 ± 0.5 Aab

**Table 3:** Mean log fold increase in HuNoV GII.1, GII.4 and GII.6 at 72 vs. 1 hpi under various treatments. Data are expressed as mean ± standard error (SE). Means with significant differences are indicated by different alphabets: capital letters indicate comparison between 1 and 72 hpi within a specific genotype and a treatment, while small letters indicate comparison of means across all treatments within either 1 or 72 hpi. HIE passages # 5–19 were defined as “Newer”, while passages 20–34 were defined as “Older”.

Fecal Processing	Serial Filtration	Serial Centrifugation			
RNA Extraction Kit	QIAamp	QIAamp	MagMAX	MagMAX	MagMAX
HIE passages #	7–28	7–28	7–28	5–19	20–34
GII.1	2.2 ± 0.5 Aa	0.9 ± 0.2 Ab	1.0 ± 0.4 Aab	1.6 ± 0.5 Aab	0.8 ± 0.4 ABab
GII.4	2.0 ± 0.6 Aa	0.4 ± 0.2 Ab	1.7 ± 0.4 Aac	2.3 ± 0.4 Aa	2.0 ± 0.5 Aa
GII.6	1.2 ± 0.4 Aa	0.9 ±0.4 Aa	1.3 ± 0.5 Aa	1.8 ± 0.6 Aa	0.4 ± 0.2 Ba

## Data Availability

All data are shared in the manuscript.
